# A two-step iterative framework for signal and image deblurring using G-I-Nonexpansive Mappings

**DOI:** 10.1371/journal.pone.0353844

**Published:** 2026-07-22

**Authors:** Esra Yolacan

**Affiliations:** Department of Information Systems and Technologies, Cappadocia University, School of Applied Sciences, Nevşehir, Türkiye; Communication University of Zhejiang, CHINA

## Abstract

This study introduces *G-I-*nonexpansive mapping by combining *I-*nonexpansive mapping with a directed graph. It also establishes convergence results for a two-step Ishikawa-type iteration. Numerical experiments were conducted on benchmark image deblurring problems, in which images were degraded by motion blur and additive Gaussian noise. The proposed method achieves competitive restoration performance, with peak signal-to-noise ratio values of up to 24.51 dB. It outperforms classical approaches such as Wiener filtering, Lucy-Richardson and the Fast Iterative Shrinkage-Thresholding Algorithm while remaining comparable to Total variation (TV)-based methods. The method reliably enhances signals in 1D, achieving a peak signal-to-noise ratio of 29.73 dB and high structural similarity index measure values. These results suggest that the framework is an effective tool for restoring signals and images degraded by blur and noise.

## 1. Introduction

Over the past century, fixed point theory has evolved into a vital tool for analysing nonlinear problems. Its mathematical foundations support a remarkably broad spectrum of applications, ranging from engineering fields such as fluid and elastic mechanics and signal processing to image reconstruction, and extending to economics and the social sciences via game theory and the concept of Nash equilibrium. Furthermore, this theory plays a central role in computer science, particularly in optimisation, iterative algorithms, and data flow analysis. It has also proven invaluable in control theory and various branches of pure mathematics [1–7].

Let Φ be a nonvoid subset of Banach space Υ, and let C: Φ→Φ be a self-mapping. The set of fixed points of C is defined by


$$\begin{array}{c} S_{C}=\left\{\varsigma \in \Phi :\text{C}(\varsigma )=\varsigma \right\}. \end{array}$$

A wide range of iterative procedures for approximating the fixed points of non-linear operators has been proposed in the literature. One of the most well-known methods is Picard iteration [8] which starts from an initial point $x_{\text{1}}\in \Upsilon$ by


$$\begin{array}{c} x_{n+\text{1}}=Cx_{n},\;\forall n\geq \text{1}. \end{array}$$

The Mann iteration [9] is generated from an initial point $x_{\text{1}}\in \Upsilon$ by


$$\begin{array}{c} x_{n+\text{1}}=\left(\text{1}-\sigma_{n}\right)x_{n}+\sigma_{n}Cx_{n},\;\forall n\geq \text{1}. \end{array}$$

where $\left\{\sigma_{n}\right\}\subset (\text{0},\text{1}).$

The Ishikawa iteration [10] is defined in a similar way, by choosing $x_{\text{1}}\in \Upsilon$ and setting


$$\begin{array}{c} x_{n+\text{1}}=\left(\text{1}-\sigma_{n}\right)x_{n}+\sigma_{n}Cy_{n}, \end{array}$$

$$\begin{array}{c} y_{n}=\left(\text{1}-\varrho_{n}\right)x_{n}+\varrho_{n}Cx_{n},\;\forall n\geq \text{1} \end{array}$$

where $\left\{\sigma_{n}\right\},\;\left\{\varrho_{n}\right\}\subset (\text{0},\text{1}).$

Of the many branches of applied mathematics, graph theory (GT) is a particularly compelling and influential field. Its wide applicability has led to a steady increase in research output over the past fifty years. Indeed, GT has been extensively employed in many disciplines, including engineering, the natural and physical sciences, genetics, computer science, sociology, operations research, economics, and linguistics [11,12]. In recent years, the study of iterative methods for approximating fixed points of mappings on abstract spaces via graphs has attracted significant attention. Jachymski [13] introduced an extension of the Banach fixed point theorem to metric spaces endowed with graphs, and also proposed the notion of a G− contraction, summarised below.

Let (Υ,d) be a complete metric space, and let G=(V,E) be a digraph with V=Υ and E containing all loops, that is,

(κ,τ)∈E⇒(Cκ,Cτ)∈E, for every κ,τ∈Υ,

and there is ω∈(0,1) such that

(κ,τ)∈E⇒d(Cκ,Cτ)≤ωd(κ,τ), for every κ,τ∈Υ.

If ω=1, then C is said to be G− nonexpansive on Υ [14].

Several contributions have been made to the literature on iterative schemes for G− nonexpansive mappings associated with graphs. In particular, authors [15] presented results on iteration algorithms for G− nonexpansive and G− contractive mappings, building on the fundamental ideas of Reich and Zaslavski. Tripak [14] further examined the convergence properties of the Ishikawa algorithm for G− nonexpansive mappings in abstract spaces equipped with a graph, and the author [16] provided additional theoretical insights into G− nonexpansive mappings. The authors [17] showed that a parallel monotone hybrid algorithm for a finite family of G− nonexpansive mappings in Hilbert spaces with graphs converges to a common fixed point and can be effectively applied to signal recovery problems. Subsequently, Khemphet et al. [18] proposed an inertial Mann-type parallel algorithm for G− nonexpansive mappings within Hilbert spaces involving digraphs. Chairatsiripong et al. [19] showed that the M− iteration method for G− nonexpansive mappings in uniformly convex Banach spaces with directed graphs converges both weakly and strongly. It also converges faster than the Noor and SP− iterations and can be effectively applied to image deblurring and signal recovery problems. Yambangwai and Thianwan [20] proposed a new computational approach for identifying common fixed points of G− nonexpansive mappings in Hilbert spaces. They proved the approach’s weak convergence and demonstrated its effectiveness in solving signal recovery problems. In 2025, Ungchittrakool and Artsawang [21] established an inertial Krasnosel’skiĭ--Mann and Ishikawa-type iterative scheme for nonexpansive mappings in Hilbert spaces. This scheme converges strongly to a fixed point and can be effectively applied to monotone inclusion and image restoration problems. More recently, Tiammee et al. [22] gave a damped double-inertial parallel algorithm with adaptive control that converges weakly to a common fixed point, achieving improved stability and faster convergence. Notably, it outperforms existing methods, including the Fast Iterative Shrinkage-Thresholding Algorithm (FISTA), in image restoration and convex feasibility problems. On the other hand, Shahzad [23] introduced a broader class of nonexpansive mappings, known as I− nonexpansive mappings, and examined the best approximation results for this class in Banach spaces. Building on this work, Rhoades and Temir [24] proved the weak convergence of Mann iterates for I− nonexpansive mappings in Banach spaces using the Opial property. Furthermore, the authors [25] developed a refined hybrid algorithm to approximate a common fixed point for a finite family of I− asymptotically nonexpansive mappings.

In this direction, motivated by the concept of g− edge preservation in the mapping C defined by the author [26], this work establishes a novel notion of G−I− nonexpansiveness, combining the concepts of graphs and I− nonexpansive mappings.

**Definition 1.1.** Let Υ be a normed space, Φ be a nonvoid subset of Υ and G=(V,E) be a digraph such that V=Φ. Let C and I be mappings from Φ to Φ. Then C is said to be G−I− nonexpansive if it satisfies the following condition for all x,y∈Υ;

i. C−I− graph preserving, i.e., (Cx,Cy)∈E⇒(Ix,Iy)∈E,

ii.  (Ix,Iy)∈E⇒‖Cx−Cy‖≤‖Ix−Iy‖.

Drawing inspiration from the work of Gunduz and Akbulut [27] and Van Dung and Trung Hieu [28], we present the following case.

**Example 1.2.** Let R=Υ be a normed space via the usual norm ‖x‖=|x| for all x∈Υ. Let Φ=[0,1] and G=(V,E) be a digraph such that Φ=V and (x,y) belongs to E if and only if 0.10000≤y≠x≤0.90000 or x=y and x,y belong to Φ. Define C:Φ→Φ and I:Φ→Φ as $Cx=\frac{x}{\text{2}}+\frac{\text{1}}{\text{4}}$ and Ix=−x+1, respectively, for all x∈Φ. Let (x,y)∈E such that 0.10000≤x,y≤0.90000. Thus, we have 0.10000≤Cx,Cy≤0.90000 and 0.10000≤Ix,Iy≤0.90000, so (Cx,Cy),(Ix,Iy)∈E. Therefore, C is an I− graph-preserving mapping. Furthermore, it is clear that C is an I− graph-preserving mapping and that I is a G− nonexpansive mapping.

**Remark 1.3.** Note that if I=Id (where Id is the identity mapping), then the G−I− nonexpansive mapping reduces to the G− nonexpansive mapping.

Let Υ be a Banach space and Φ a nonvoid subset of Υ. Consider the G−I− nonexpansive mapping C:Φ→Φ, where I:Φ→Φ is a G−nonexpansive mapping. Then for $x_{\text{1}}\in \Upsilon$, consider the following iteration method:


$$\begin{array}{c} \begin{array}{c} x_{n+\text{1}}=\left(\text{1}-\sigma_{n}\right)x_{n}+\sigma_{n}Iy_{n}\;,\\ y_{n}=\left(\text{1}-\varrho_{n}\right)x_{n}+\varrho_{n}Cx_{n},\;\forall n\geq \text{1}, \end{array} \end{array}$$ (1)

where $\left\{\sigma_{n}\right\},\;\left\{\varrho_{n}\right\}\subset (\text{0},\text{1}).$

Using method (1), we derive some convergence theorems for approximating common fixed points of G−I− nonexpansive mappings and G− nonexpansive mappings on Banach spaces endowed with a digraph. To validate these results, we present numerical experiments on benchmark image deblurring problems, as well as simulation results for 1D signal enhancement. Unlike TV-based methods, which can result in oversmoothing and the loss of fine textures, the proposed framework aims to preserve structural details while effectively suppressing noise.

## 2. Related Work

Various computational and mathematical approaches to image restoration and deblurring have been extensively studied. Classical methods such as Wiener filtering and Lucy--Richardson (LR) deconvolution are based on the principles of inverse filtering and maximum likelihood estimation [29–31]. While these methods are computationally efficient, they are susceptible to noise amplification and ringing artefacts [32], which can degrade the quality of restored images, particularly when there is a high level of noise.

To address these limitations, modern optimisation-based methods have been widely developed. In particular, total variation (TV) regularisation has proven effective in preserving edges while suppressing noise [33]. Furthermore, advanced frameworks such as the Alternating Direction Method of Multipliers (ADMM) and the Fast Iterative Shrinkage-Thresholding Algorithm (FISTA) efficiently solve large-scale inverse problems and offer improved reconstruction quality and convergence speed [34,35]. Recent studies have demonstrated the effectiveness of these methods in restoring images and solving convex feasibility problems [20–22,36]. However, these approaches typically require careful parameter tuning and can result in oversmoothing and the loss of fine image textures [34,35].

In parallel, classical iterative schemes such as the Picard [8], Mann [9] and Ishikawa [10] iterations have been extensively used to solve nonlinear operator equations. Building on these foundations, the effectiveness of G− nonexpansive mappings [14] and graph-based settings in signal recovery and image processing applications [19,22] has been demonstrated. These approaches provide a robust theoretical basis for designing iterative algorithms with guaranteed convergence.

Recently, deep learning-based approaches have achieved remarkable success in image restoration tasks, particularly deblurring and denoising. Fayolle and Belyaev [37] introduced variants of the modified Richardson-Lucy (RL) and Image Field Reconstruction (ISRA) algorithm that accelerate convergence and improve restoration quality. This was achieved by interpreting these methods as fixed-point iterations within a variational framework and incorporating adaptive image smoothing. Aberdeen et al. [38] demonstrated the effectiveness of U-Net-based deep learning frameworks for recovering blurred images of resident space objects (RSOs), improving reconstruction quality and pose estimation accuracy. Hu et al. [39] proposed a lightweight deep learning framework combining a MobileNetV2 backbone with multi-scale transformer modelling and channel attention mechanisms to achieve robust performance in industrial scenarios. Additionally, Rong and Huang [40] developed a hybrid deblurring method integrating an unsupervised encoder-decoder network with an optimisation framework. This method provides improved restoration quality and generalisation without requiring labelled training data. However, deep learning-based methods typically require substantial training datasets and considerable computational resources, while optimisation-based approaches often necessitate precise parameter tuning and can result in the loss of fine structural details.

By contrast, this study puts forward a G−I− nonexpansive mapping framework that extends classical fixed-point theory to graph-structured settings. The proposed method combines theoretical convergence guarantees with practical applicability, delivering restoration performance that rivals Wiener filtering, LR and FISTA in both image deblurring and 1D signal enhancement.

## 3. Preliminaries

In this section, we establish the lemmas and definitions that will be used in the fourth part. From now on, we will show the strong and weak convergence of $\left\{x_{n}\right\}$ to a point ς∈Φ as $x_{n}\to \varsigma$ and $x_{n}⇀\varsigma$, respectively.

In the sequel, we assume that Υ is a Banach space and G is a digraph. In addition, suppose that G holds no parallel edges, and thus we could describe G=(V,E). A digraph G is said an *oriented graph* if whenever (κ,τ)∈E, then (τ,κ)∉E. If κ,τ are vertices of G, then a *directed path* from κ to τ of length M is a sequence ${\left\{\kappa_{i}\right\}}_{i=\text{0}}^{M}$ of M+1 vertices such that $\kappa =\kappa_{\text{0}},$ $\tau =\kappa_{M}$ and $\left(\kappa_{i-\text{1}},\kappa_{i}\right)\in E$ for $i=\overset{―}{\text{1},M}.$ G is called to be *connected* if there is a path among any two vertices. A digraph G is called to be *transitive* if, (κ,τ),(τ,ς)∈E for ∀κ,τ,ς ∈V, we acquire (κ,ς)∈E. GT terminology and notations are standard and can be found in any GT books (for details see [41–43]).

**Definiton 3.1** [44]. Let Υ be a normed space, Φ be a nonvoid subset of Υ, and G=(V,E) be a digraph such that V=Φ. Then Φ is said to satisfy property (G) if for any sequence $\left\{\kappa_{n}\right\}\subseteq \Phi$ such that $\left(\kappa_{n},\kappa_{n+\text{1}}\right)\in E$ and $\left\{\kappa_{n}\right\}⇀\kappa \in \Phi$, there is a subsequence $\left\{\kappa_{l(n)}\right\}$ of $\left\{\kappa_{n}\right\}$ such that $\left(\kappa_{l(n)},\kappa \right)\in E$ for every n∈N.

**Definiton 3.2** [45]. Let Υ be a normed space, Φ be a nonvoid subset of Υ, and C:Φ→Φ be a mapping. Then C is said to be semi-compact if for $\left\{\kappa_{n}\right\}\subseteq \Phi$ with $\underset{n\to \infty }{\text{lim}}\left\|\kappa_{n}-C\kappa_{n}\right\|=\text{0},$ there appears a subsequence $\left\{\kappa_{n_{i}}\right\}$ of $\left\{\kappa_{n}\right\}$ such that $\kappa_{n_{i}}\to \varsigma \in \Phi .$

**Definiton 3.3** [46]. The mappings C,I:Φ→Φ are said to satisfy condition (A) if there is a nondecreasing function Ω:[0,∞)→[0,∞) with Ω(0)=0 and Ω(t)>0 for ∀t∈[0,∞) such that $\frac{\text{1}}{\text{2}}\left(\left\|\kappa -C\kappa \right\|+\left\|\kappa -I\kappa \right\|\right)\geq \Omega \left(d(\kappa ,S)\right)$ for every κ∈Φ, $d(\kappa ,S)=inf\left\{\left\|\kappa -\varsigma \right\|:\varsigma \in S=S_{C}⋂S_{I}\right\}.$

**Definiton 3.4** [47]. A Banach space Υ is said to have the Opial property if, for all sequences $\left\{\kappa_{n}\right\}\subseteq \Upsilon$ such that $\left\{\kappa_{n}\right\}⇀\kappa \in \Upsilon$, the inequality $\underset{n⟶\infty }{\text{lim}}sup\left\|\kappa_{n}-\kappa \right\|<\underset{n⟶\infty }{\text{lim}}sup\left\|\kappa_{n}-\tau \right\|$ holds for all τ≠κ in Υ.

**Definiton 3.5** [48]. Let Φ be a nonvoid subset of a Banach space Υ, and C:Φ→Υ be a mapping. Then, C is said to be G− demiclosed at τ∈Υ if, for any sequence $\left\{\kappa_{n}\right\}\subseteq \Upsilon$ such that $\left\{\kappa_{n}\right\}⇀\kappa \in \Upsilon$, $\left\{C\kappa_{n}\right\}\to \tau$ and $\left(\kappa_{n},\kappa_{n+\text{1}}\right)\in E$ imply Cκ=τ.

**Definiton 3.6** [28]**.** Let Υ be a vector space and L be a nonvoid subset of Υ×Υ. Then L is said to be coordinate-convex if for all (q,τ), (q,κ),(τ,q),(κ,q)∈L and for ∀ξ∈[0,1], we hold


$$\begin{array}{c} \xi (q,\tau )+(\text{1}-\xi )(q,\kappa )\in L\text{ and }\xi (\tau ,q)+(\text{1}-\xi )(\kappa ,q)\in L. \end{array}$$

**Lemma 3.7** [49]. Let Υ be a Banach space satisfy the Opial property, $\left\{\kappa_{n}\right\}\subseteq \Phi ,\underset{n\to \infty }{\text{lim}}\left\|\kappa_{n}-\kappa \right\|$ and $\underset{n\to \infty }{\text{lim}}\left\|\kappa_{n}-\tau \right\|$ exist for some κ,τ∈Υ, and there exist $\left\{\kappa_{l(n)}\right\}$ and $\left\{\tau_{l(n)}\right\}\;$be two subsequences of $\left\{\kappa_{n}\right\}$ which weakly converge to κ and τ, respectively. Then κ=τ.

**Lemma 3.8** [50]**.** Let Υ be a uniformly convex Banach space and let m,n be two constants with 0<m<n<1. Assume that $\left\{a_{n}\right\}\subset [m,n]$ is a real sequence and $\left\{\kappa_{n}\right\},$ $\left\{\tau_{n}\right\}\subseteq \Upsilon .$ Then the terms


$$\begin{array}{c} \underset{n\to \infty }{\text{lim}}\left\|{a_{n}\kappa }_{n}+(\text{1}-a_{n})\tau_{n}\right\|=e, \end{array}$$

$$\begin{array}{c} \underset{n⟶\infty }{\text{lim}}sup\left\|\kappa_{n}\right\|\leq e, \end{array}$$

$$\begin{array}{c} \underset{n⟶\infty }{\text{lim}}sup\left\|\tau_{n}\right\|\leq e, \end{array}$$

imply that $\underset{n\to \infty }{\text{lim}}\left\|\kappa_{n}-\tau_{n}\right\|=\text{0},$ where e≥0 is a constant.

## 4. Main results

In this section, we demonstrate some of the features of (1), which represent the progress of the outcomes in Tripak [14] by using coordinate convexity instead of the convexity of E.

**Proposition 4.1.** Assume that Υ is a normed space, and Φ is a nonvoid convex subset of Υ. G=(V,E) is a digraph and transitive such that V=Φ and E is coordinate-convexity. C:Φ→Φ is I− edge preserving, where I: Φ→Φ is edge preserving and $S=S_{C}⋂S_{I}.$ For each ς∈S, the sequence $\left\{x_{n}\right\}$ is described as in (1) such that $\left(x_{\text{1}},\varsigma \right),\left(\varsigma ,x_{\text{1}}\right)\in E.$ Then $\left(\varsigma ,x_{n}\right),$ $\left(x_{n},\varsigma \right)$, $\left(\varsigma ,y_{n}\right),$ $\left(y_{n},\varsigma \right)$, $\left(y_{n},x_{n}\right)$, $\left(x_{n},y_{n}\right)$, $\left(x_{n},x_{n+\text{1}}\right)\in E$ for every n∈N.

*Proof:* Initially, we verify inductively that $\left(\varsigma ,x_{n}\right)\in E.$ Clearly, $\left(\varsigma ,x_{\text{1}}\right)\in E.$ Next, assume that $\left(\varsigma ,x_{n}\right)\in E$ belongs to n=k. We will demonstrate $\left(\varsigma ,x_{k+\text{1}}\right)\in E.$ In fact, as C is I− edge preserving, we get $\left(\varsigma ,{Cx}_{k}\right)\in E.$ Note that


$$\begin{array}{c} \left(\varsigma ,y_{k}\right)=\left(\varsigma ,\left(\text{1}-\varrho_{k}\right)x_{k}+\varrho_{k}Cx_{k}\right)=\left(\text{1}-\varrho_{k}\right)\left(\varsigma ,x_{k}\right)+\varrho_{k}\left(\varsigma ,Cx_{k}\right). \end{array}$$ (2)

Due to the coordinate-convexity of E and adding (2) with $\left(\varsigma ,x_{k}\right),$ $\left(\varsigma ,Cx_{k}\right)\in E$, we obtain $\left(\varsigma ,y_{k}\right)\in E.$ Then, since I is edge preserving, we get $\left(\varsigma ,Iy_{k}\right)\in E$. Indeed,


$$\begin{array}{c} \left(\varsigma ,x_{k+\text{1}}\right)=\left(\varsigma ,\left(\text{1}-\sigma_{k}\right)x_{k}+\sigma_{k}Iy_{k}\right)=\left(\text{1}-\sigma_{k}\right)\left(\varsigma ,x_{k}\right)+\sigma_{k}\left(\varsigma ,Iy_{k}\right). \end{array}$$ (3)

Again, by the coordinate-convexity of E and equation (3), we get $\left(\varsigma ,x_{k+\text{1}}\right)\in E$. This shows that $\left(\varsigma ,x_{n}\right)\in E.$

Next, we show that $\left(\varsigma ,x_{n}\right)\in E.$ Because C is I−edge preserving, we have $\left(\varsigma ,{Cx}_{n}\right)\in E.\;$Moreover,


$$\begin{array}{c} \left(\varsigma ,y_{n}\right)=\left(\varsigma ,\left(\text{1}-\varrho_{n}\right)x_{n}+\varrho_{n}Cx_{n}\right)=\left(\text{1}-\varrho_{n}\right)\left(\varsigma ,x_{n}\right)+\varrho_{n}\left(\varsigma ,Cx_{n}\right). \end{array}$$ (4)

Owing to the coordinate-convexity of E and adding (4) with $\left(\varsigma ,x_{n}\right)$, $\left(\varsigma ,Cx_{n}\right)\in E$, we obtain $\left(\varsigma ,y_{n}\right)\in E$. Therefore, I is edge preserving, we have $\left(\varsigma ,{Iy}_{n}\right)\in E$. To be clear,


$$\begin{array}{c} \left(\varsigma ,x_{n+\text{1}}\right)=\left(\varsigma ,\left(\text{1}-\sigma_{n}\right)x_{n}+\sigma_{n}Iy_{n}\right)=\left(\text{1}-\sigma_{n}\right)\left(\varsigma ,x_{n}\right)+\sigma_{n}\left(\varsigma ,Iy_{n}\right). \end{array}$$ (5)

Again, by the coordinate-convexity of E and equation (5), we get $\left(\varsigma ,x_{n+\text{1}}\right)\in E$. Using a similar argument, we conclude that $\left(x_{n},\varsigma \right)$, $\left(y_{n},\varsigma \right)\in E$ for every n∈N. As G is transitive, $\left(x_{n},\varsigma \right)$, $\left(\varsigma ,y_{n}\right)$, $\left(y_{n},\varsigma \right)$, $\left(\varsigma ,x_{n}\right)\in E$, we acquire $\left(y_{n},x_{n}\right)$, $\left(x_{n},y_{n}\right)$, $\left(x_{n},x_{n+\text{1}}\right)\in E$ for every n∈N.

**Lemma 4.2.** Assume that Υ is a normed space, and Φ is a nonvoid closed convex subset of Υ. G=(V,E) is a digraph and transitive such that V=Φ and E is coordinate-convexity. G−I− nonexpansive mapping C:Φ→Φ, where I:Φ→Φ is G− nonexpansive mapping and $S=S_{C}⋂S_{I}.$ For each ς∈S, the sequence $\left\{x_{n}\right\}$ is described as in (1) such that $\left(x_{\text{1}},\varsigma \right),\left(\varsigma ,x_{\text{1}}\right)\in E\;$and $\left\{\sigma_{n}\right\}$ and $\left\{\varrho_{n}\right\}\subset [\varphi ,\text{1}-\varphi ]$ for some φ∈(0,1). Then

i. $\left\{x_{n}\right\}$ is bounded and $\underset{n\to \infty }{\text{lim}}\left\|x_{n}-\varsigma \right\|$ exists;
ii. $\underset{n\to \infty }{\text{lim}}\left\|x_{n}-Cx_{n}\right\|=\underset{n\to \infty }{\text{lim}}\left\|x_{n}-Ix_{n}\right\|=\text{0}.$

*Proof:* i. Let ς∈S and $\left(x_{\text{1}},\varsigma \right),\left(\varsigma ,x_{\text{1}}\right)\in E.$ From Proposition 4.1, we get $\left(\varsigma ,x_{n}\right),$ $\left(x_{n},\varsigma \right)$, $\left(\varsigma ,y_{n}\right),$ $\left(y_{n},\varsigma \right)$, $\left(y_{n},x_{n}\right)$, $\left(x_{n},y_{n}\right)$, $\left(x_{n},x_{n+\text{1}}\right)\in E$ for every n∈N. Using G−I− nonexpansive mapping C:Φ→Φ, where I:Φ→Φ is G− nonexpansive mapping, we obtain


$$\begin{array}{c} \left\|y_{n}-\varsigma \right\|\leq \left(\text{1}-\varrho_{n}\right)\left\|x_{n}-\varsigma \right\|+\varrho_{n}\left\|Cx_{n}-\varsigma \right\| \end{array}$$ (6)

$$\begin{array}{c} \leq \left(\text{1}-\varrho_{n}\right)\left\|x_{n}-\varsigma \right\|+\varrho_{n}\left\|Ix_{n}-\varsigma \right\| \end{array}$$

$$\begin{array}{c} \leq \left\|x_{n}-\varsigma \right\|. \end{array}$$

Using (6) and G− nonexpansive mapping of I, we get


$$\begin{array}{c} \left\|x_{n+\text{1}}-\varsigma \right\|\leq \left(\text{1}-\sigma_{n}\right)\left\|x_{n}-\varsigma \right\|+\sigma_{n}\left\|Iy_{n}-\varsigma \right\| \end{array}$$ (7)

$$\begin{array}{c} \leq \left(\text{1}-\sigma_{n}\right)\left\|x_{n}-\varsigma \right\|+\sigma_{n}\left\|y_{n}-\varsigma \right\| \end{array}$$

$$\begin{array}{c} \leq \left\|x_{n}-\varsigma \right\|. \end{array}$$

From inequality (7), it follows that the sequence $\left\{x_{n}\right\}$ is bounded and the limit $\underset{n\to \infty }{\text{lim}}\left\|x_{n}-\varsigma \right\|$ exists.

ii. Let ς∈S and $\left(x_{\text{1}},\varsigma \right),\left(\varsigma ,x_{\text{1}}\right)\in E.$ From Proposition 4.1, we have that $\left(\varsigma ,x_{n}\right),$ $\left(x_{n},\varsigma \right)$, $\left(\varsigma ,y_{n}\right),$ $\left(y_{n},\varsigma \right)$, $\left(y_{n},x_{n}\right)$, $\left(x_{n},y_{n}\right)$, $\left(x_{n},x_{n+\text{1}}\right)\in E$ for every n∈N. From Lemma 4.2 (i), $\underset{n\to \infty }{\text{lim}}\left\|x_{n}-\varsigma \right\|$ exists. Put $\underset{n\to \infty }{\text{lim}}\left\|x_{n}-\varsigma \right\|=e$.

Letting limsup on both sides in (6),


$$\begin{array}{c} \underset{n\to \infty }{\text{lim}}sup\left\|y_{n}-\varsigma \right\|\leq e. \end{array}$$

Since I is G− nonexpansive mapping, we can get that


$$\begin{array}{c} \underset{n\to \infty }{\text{lim}}sup\left\|Iy_{n}-\varsigma \right\|\leq e. \end{array}$$

Moreover, from (1), we have


$$\begin{array}{c} \left(x_{n+\text{1}}-\varsigma \right)=\left(\text{1}-\sigma_{n}\right)\left(x_{n}-\varsigma \right)+\sigma_{n}\left(Iy_{n}-\varsigma \right). \end{array}$$

Taking the limit as n→∞ on both sides yields:


$$\begin{array}{c} \underset{n\to \infty }{\text{lim}}\left\|x_{n+\text{1}}-\varsigma \right\|=\underset{n\to \infty }{\text{lim}}\left\{\left(\text{1}-\sigma_{n}\right)\left\|x_{n}-\varsigma \right\|+\sigma_{n}\left\|Iy_{n}-\varsigma \right\|\right\}=e. \end{array}$$

By Lemma 3.8, we get


$$\begin{array}{c} \underset{n\to \infty }{\text{lim}}\left\|Iy_{n}-x_{n}\right\|=\text{0}. \end{array}$$ (8)

Further, from (1), we obtain


$$\begin{array}{c} \left\|x_{n+\text{1}}-x_{n}\right\|\leq \sigma_{n}\left\|Iy_{n}-x_{n}\right\|. \end{array}$$

Hence, by (8), we attain


$$\begin{array}{c} \underset{n\to \infty }{\text{lim}}\left\|x_{n+\text{1}}-x_{n}\right\|=\text{0}. \end{array}$$

Next,


$$\begin{array}{c} \left\|x_{n}-\varsigma \right\|\leq \left\|x_{n}-Iy_{n}\right\|+\left\|Iy_{n}-\varsigma \right\|\leq \left\|x_{n}-Iy_{n}\right\|+\left\|y_{n}-\varsigma \right\| \end{array}$$

which on taking the limit as n→∞ implies


$$\begin{array}{c} \underset{n\to \infty }{\text{lim}}sup\left\|y_{n}-\varsigma \right\|=e. \end{array}$$

Given that C is G−I− nonexpansive mapping and I is G− nonexpansive mapping, we get


$$\begin{array}{c} \left\|{Cx}_{n}-\varsigma \right\|\leq \left\|Ix_{n}-\varsigma \right\|\leq \left\|x_{n}-\varsigma \right\|. \end{array}$$ (9)

Letting limsup on both side in (9),


$$\begin{array}{c} \underset{n\to \infty }{\text{lim}}sup\left\|{Cx}_{n}-\varsigma \right\|\leq e. \end{array}$$

Moreover, from (1), we have


$$\begin{array}{c} \left(y_{n}-\varsigma \right)=\left(\text{1}-\varrho_{n}\right)\left(x_{n}-\varsigma \right)+\varrho_{n}\left(Cx_{n}-\varsigma \right). \end{array}$$

Taking the limit as n→∞ on both sides yields:


$$\begin{array}{c} \underset{n\to \infty }{\text{lim}}\left\|y_{n}-\varsigma \right\|=\underset{n\to \infty }{\text{lim}}\left\{\left(\text{1}-\varrho_{n}\right)\left\|x_{n}-\varsigma \right\|+\varrho_{n}\left\|Cx_{n}-\varsigma \right\|\right\}=e. \end{array}$$

Due to Lemma 3.8, we hold


$$\begin{array}{c} \underset{n\to \infty }{\text{lim}}\left\|{Cx}_{n}-x_{n}\right\|=\text{0}. \end{array}$$ (10)

Further, we obtain


$$\begin{array}{c} \left\|{Ix}_{n}-x_{n}\right\|\leq \left\|{Ix}_{n}-{Iy}_{n}\right\|+\left\|{Iy}_{n}-x_{n}\right\| \end{array}$$

$$\begin{array}{c} \leq \left\|x_{n}-y_{n}\right\|+\left\|{Iy}_{n}-x_{n}\right\| \end{array}$$

$$\begin{array}{c} \leq \left\|{Cx}_{n}-x_{n}\right\|+\left\|{Iy}_{n}-x_{n}\right\|. \end{array}$$

Consequently, combining (8) and (10), we find that


$$\begin{array}{c} \underset{n\to \infty }{\text{lim}}\left\|{Ix}_{n}-x_{n}\right\|=\text{0}. \end{array}$$ (11)

The proof is complete.

Motivated by Suparatulatorn et al. [48] we give the following Proposition 4.3.

**Proposition 4.3.** Assume that Υ is a Banach space providing Opial’s condition, Φ is a nonvoid subset of Υ and Φ hold property (G). G=(V,E) is digraph such that V=Φ. G−I− nonexpansive mapping C:Φ→Φ, where I: Φ→Φ is G− nonexpansive mapping. C provides the property Definition 3.5 and Lemma 4.2. Then $C\varsigma_{*}=\varsigma_{*}.$

*Proof:* Assume that $\Phi ⊇\left\{x_{n}\right\}⇀\varsigma_{*}$ with $\left(x_{n},x_{n+\text{1}}\right)\in E$ and $\underset{n\to \infty }{\text{lim}}\left\|x_{n}-Cx_{n}\right\|=\underset{n\to \infty }{\text{lim}}\left\|x_{n}-Ix_{n}\right\|=\text{0}.$ Using the property (G), there is a subsequence $\left\{x_{n_{j}}\right\}$ of $\left\{x_{n}\right\}$ such that $\left(x_{n_{j}},\varsigma_{*}\right)\in E$ for every j∈N. Assume for contradiction that $C\varsigma_{*}\neq \varsigma_{*}$. By the Opial property, we conclude that


$$\begin{array}{c} \underset{n\to \infty }{\text{lim}}sup\left\|x_{n_{j}}-\varsigma_{*}\right\|\leq \underset{n\to \infty }{\text{lim}}sup\left\|x_{n_{j}}-C\varsigma_{*}\right\| \end{array}$$

$$\begin{array}{c} \leq \underset{n\to \infty }{\text{lim}}sup\left(\left\|x_{n_{j}}-Cx_{n_{j}}\right\|+\left\|Cx_{n_{j}}-C\varsigma_{*}\right\|\right) \end{array}$$

$$\begin{array}{c} \leq \underset{n\to \infty }{\text{lim}}sup\left(\left\|x_{n_{j}}-Cx_{n_{j}}\right\|+\left\|Ix_{n_{j}}-I\varsigma_{*}\right\|\right) \end{array}$$

$$\begin{array}{c} \leq \underset{n\to \infty }{\text{lim}}sup\left\|x_{n_{j}}-\varsigma_{*}\right\|. \end{array}$$

This is a contradiction. Therefore, $C\varsigma_{*}=\varsigma_{*}.$

**Theorem 4.4.** Assume that Υ is a uniformly convex Banach space providing Opial property, Φ is a nonvoid closed convex subset of Υ and Φ hold property (G). G=(V,E) is a digraph and transitive such that V=Φ and E is coordinate-convexity. G−I− nonexpansive mapping C:Φ→Φ, where I: Φ→Φ is G− nonexpansive mapping and $S=S_{C}⋂S_{I}.$ The sequence $\left\{x_{n}\right\}$ is described as in (1) such that $\left(x_{\text{1}},\varsigma \right),\left(\varsigma ,x_{\text{1}}\right)\in E\;$and $\left\{\sigma_{n}\right\}$ and $\left\{\varrho_{n}\right\}\subset [\varphi ,\text{1}-\varphi ]$ for some φ∈(0,1). Then $x_{n}⇀\varsigma_{*}\in S.$

*Proof:* As Υ is a uniformly convex Banach space, we have that Υ is a reflexive Banach space. Furthermore, as in Lemma 4.2 (i), it follows $\left\{x_{n}\right\}$ is bounded. Then there is a subsequence $\left\{x_{n_{j}}\right\}$ of $\left\{x_{n}\right\}$ such that $\left\{x_{n_{j}}\right\}⇀\varsigma_{*}\in \Phi .$ From Lemma 4.2 (ii), we also obtain


$$\begin{array}{c} \underset{n\to \infty }{\text{lim}}\left\|Cx_{n_{j}}-x_{n_{j}}\right\|=\underset{n\to \infty }{\text{lim}}\left\|Ix_{n_{j}}-x_{n_{j}}\right\|=\text{0}. \end{array}$$

By Proposition 4.3, we conclude that $C\varsigma_{*}=I\varsigma_{*}=\varsigma_{*},$ and so $\varsigma_{*}\in S.$

Assume that there is a subsequence $\left\{x_{n_{k}}\right\}$ of $\left\{x_{n}\right\}$ such that $\left\{x_{n_{k}}\right\}⇀\tau_{*}\in \Phi$ with $\varsigma_{*}\neq \tau_{*}.$ Using Proposition 4.3 and a similar argument, we deduce that $\tau_{*}\in S.$ By Lemma 4.2 (i), $\underset{n\to \infty }{\text{lim}}\left\|x_{n}-\varsigma_{*}\right\|$ and $\underset{n\to \infty }{\text{lim}}\left\|x_{n}-\tau_{*}\right\|$ exists. From Lemma 3.7, we obtain that $\varsigma_{*}=\tau_{*}.$ Thus $x_{n}⇀\varsigma_{*}\in S.$

**Proposition 4.5.** Suppose that Υ is a normed space and Φ is a nonvoid subset of Υ providing property (G). G=(V,E) is a digraph such that V=Φ and E is convex. Assume G−I− nonexpansive mapping C:Φ→Φ, where I: Φ→Φ is G− nonexpansive mapping and $S_{C}\times S_{C}\subset E$ and $S_{I}\times S_{I}\subset E$. Then S is closed and convex.

*Proof:* Use the line of procedure endowed in the proof of Theorem 3.2 in [44], we can easily see that S is closed and convex.

**Theorem 4.6.** Assume that Υ is a uniformly convex Banach space providing Opial property, Φ is a nonvoid closed convex subset of Υ and Φ hold property (G). G=(V,E) is a digraph and transitive such that V=Φ and E is coordinate-convexity. G−I− nonexpansive mapping and C:Φ→Φ, where I: Φ→Φ is G−nonexpansive mapping such that $S=S_{C}⋂S_{I}$, $S_{C}\times S_{C}\subset E$ and $S_{I}\times S_{I}\subset E$ and C, I provide the condition (A). The sequence$\;\left\{x_{n}\right\}$ is described as in (1) such that $\left(x_{\text{1}},\varsigma \right),\left(\varsigma ,x_{\text{1}}\right)\in E\;$and $\left\{\sigma_{n}\right\}$ and $\left\{\varrho_{n}\right\}\subset [\varphi ,\text{1}-\varphi ]$ for some φ∈(0,1). Then $x_{n}\to \varsigma \in S.$

*Proof:* Let ς∈S and $\left(x_{\text{1}},\varsigma \right),\left(\varsigma ,x_{\text{1}}\right)\in E.$ From Proposition 4.1, we get $\left(\varsigma ,x_{n}\right),$ $\left(x_{n},\varsigma \right)$, $\left(\varsigma ,y_{n}\right),$ $\left(y_{n},\varsigma \right)$, $\left(y_{n},x_{n}\right)$, $\left(x_{n},y_{n}\right)$, $\left(x_{n},x_{n+\text{1}}\right)\in E$ for every n∈N. From Lemma 4.2 (i) and (7),


$$\begin{array}{c} \left\|x_{n+\text{1}}-\varsigma \right\|\leq \left\|x_{n}-\varsigma \right\|. \end{array}$$

This implies that


$$\begin{array}{c} d\left(x_{n+\text{1}},S\right)\leq d\left(x_{n},S\right), \end{array}$$

and so, $d\left(x_{n},S\right)\;$exists. Also by Lemma 4.2 (ii), we get


$$\begin{array}{c} \underset{n\to \infty }{\text{lim}}\left\|x_{n}-Cx_{n}\right\|=\underset{n\to \infty }{\text{lim}}\left\|x_{n}-Ix_{n}\right\|=\text{0}. \end{array}$$

The condition (A) guarantees that $\underset{n\to \infty }{\text{lim}}\Omega \left(d\left(x_{n},S\right)\right)=\text{0}.$ As Ω is a nondecreasing function and Ω(0)=0, it follows that $\underset{n\to \infty }{\text{lim}}d\left(x_{n},S\right)=\text{0}.$ Thus, we may receive a subsequence $\left\{x_{n_{j}}\right\}$ of $\left\{x_{n}\right\}$ and a sequence $\left\{\varsigma_{n}\right\}\subseteq S$ such that


$$\begin{array}{c} \left\|x_{n_{j}}-\varsigma_{n}\right\|<{\text{2}}^{-j}. \end{array}$$ (12)

Using the proof procedure of [51], we have


$$\begin{array}{c} \left\|x_{n_{j+\text{1}}}-\varsigma_{n}\right\|\leq \left\|x_{n_{j}}-\varsigma_{n}\right\|<{\text{2}}^{-j}. \end{array}$$

Thus,


$$\begin{array}{c} \left\|\varsigma_{n+\text{1}}-\varsigma_{n}\right\|\leq \left\|\varsigma_{n+\text{1}}-x_{n_{j+\text{1}}}\right\|+\left\|x_{n_{j+\text{1}}}-\varsigma_{n}\right\|\leq {\text{2}}^{-j+\text{1}}. \end{array}$$

We deduce that $\left\{\varsigma_{n}\right\}$ is a Cauchy sequence in S. By Proposition 4.5, S is closed. Hence, there exists ς∈S such that


$$\begin{array}{c} \underset{n\to \infty }{\text{lim}}\varsigma_{n}=\varsigma . \end{array}$$ (13)

Combining expressions (12) and (13), we conclude that $\underset{n\to \infty }{\text{lim}}\left\|x_{n_{j}}-\varsigma \right\|=\text{0}$. Hence, by Lemma 4.2 (i), we conclude that $x_{n}\to \varsigma \in S.$

**Theorem 4.7.** Under the presumptions of Theorem 4.6, if either C or I is semi-compact, then $\left\{x_{n}\right\}\to \varsigma \in S.$

*Proof:* From Lemma 4.2 (i) and (ii), we obtain that $\left\{x_{n}\right\}$ is bounded and $\underset{n\to \infty }{\text{lim}}\left\|x_{n}-Cx_{n}\right\|=\underset{n\to \infty }{\text{lim}}\left\|x_{n}-Ix_{n}\right\|=\text{0}.$ Since either C or I is semi-compact, there is a subsequence $\left\{x_{n_{j}}\right\}$ of $\left\{x_{n}\right\}$ such that $\left\{x_{n_{j}}\right\}\to \tau^{*}\in \Phi .$ Because G is transitive and Φ has property (G), there exists a subsequence $\left\{x_{n_{j}}\right\}$ of $\left\{x_{n}\right\}$ such that $\left(x_{n_{j}},\varsigma \right)\in E.$ Notice that


$$\begin{array}{c} \left\|\varsigma -C\varsigma \right\|\leq \left\|\varsigma -x_{n_{j}}\right\|+\left\|x_{n_{j}}-{Cx}_{n_{j}}\right\|+\left\|{Cx}_{n_{j}}-C\varsigma \right\|, \end{array}$$ (14)

$$\begin{array}{c} \left\|\varsigma -I\varsigma \right\|\leq \left\|\varsigma -x_{n_{j}}\right\|+\left\|x_{n_{j}}-{Ix}_{n_{j}}\right\|+\left\|{Ix}_{n_{j}}-I\varsigma \right\|. \end{array}$$ (15)

Taking the limit as J→∞ in (14) and (15), we attain ς=Cς, ς=Iς which means ς∈S. After all, the limit $\underset{n\to \infty }{\text{lim}}\left\|x_{n}-\varsigma \right\|$ exists owing to Lemma 4.2 (i); thus,


$$\begin{array}{c} \underset{n\to \infty }{\text{lim}}\left\|x_{n}-\varsigma \right\|=\text{0}=\underset{n\to \infty }{\text{lim}}\left\|x_{n_{j}}-\varsigma \right\| \end{array}$$

which concludes that $\left\{x_{n}\right\}\to \varsigma \in S.$ The proof is complete.

To demonstrate the validity of Theorem 4.7, we will apply the numerical example given below.

**Example 4.8.** Let R=Υ be a normed space via the usual norm ‖x‖=|x| for every x∈Υ, Φ=[0,1] and G=(V,E) be a digraph such that Φ=V and (x,y)∈E if and only if 0.10000≤y≠x≤0.90000 or x=y∈Φ. Here, E is coordinate-convexity and E⊃{(x,x):x∈V}. Define C, I:Φ→Φ as $Cx=\frac{x}{\text{2}}+\frac{\text{1}}{\text{4}}$ and Ix=−x+1 for every x∈Φ. Let (x,y)∈E, we reckon with 0.10000≤x,y≤0.90000. Therefore, we have 0.10000≤Cx,Cy≤0.90000 and 0.10000≤Ix,Iy≤0.90000, so (Cx,Cy),(Ix,Iy)∈E. Then, C is I− graph preserving. Furthermore, it is clear that C is G−I− nonexpansive mapping and I is G− nonexpansive mapping. We also have S={0.50000}. Moreover, C and I are semi-compact. Set $\sigma_{n}=\frac{\text{3}n+\text{1}\text{3}}{\text{7}n+\text{1}\text{7}}$ and $\varrho_{n}=\frac{\text{8}n+\text{1}\text{5}}{\text{1}\text{1}n+\text{3}\text{2}}.$ Therefore, all the assumptions of Theorem 4.7 are met. Next, we indicate that $x_{n}\to \text{0}.\text{5}\text{0}\text{0}\text{0}\text{0}\in S.$ Taking n=1 and $x_{\text{0}}\in \Phi ,$ we obtain $\sigma_{\text{0}}=\frac{\text{1}\text{3}}{\text{1}\text{7}}$ and $\varrho_{\text{0}}=\frac{\text{1}\text{5}}{\text{3}\text{2}}$ and get $x_{\text{1}}$ from


$$\begin{array}{c} \begin{array}{c} x_{\text{1}}=\left(\text{1}-\sigma_{\text{0}}\right)x_{\text{0}}+\sigma_{\text{0}}Iy_{\text{0}}\;,\\ y_{\text{0}}=\left(\text{1}-\varrho_{\text{0}}\right)x_{\text{0}}+\varrho_{\text{0}}Cx_{\text{0}}.\; \end{array} \end{array}$$

Similarly, $x_{\text{2}},\;x_{\text{3}},\cdots ,x_{n},\cdots$. All computational procedures were executed using MATLAB R2016a. We give the first five values of $\left\{x_{n}\right\}$ as in the Table 1 below for the initial term $x_{\text{0}}=\text{0}.\text{1}\text{0}\text{0}\text{0}\text{0},$ $x_{\text{0}}=\text{0}.\text{3}\text{0}\text{0}\text{0}\text{0},$ $x_{\text{0}}=\text{0}.\text{7}\text{0}\text{0}\text{0}\text{0}$ and $x_{\text{0}}=\text{0}.\text{9}\text{0}\text{0}\text{0}\text{0},$ respectively. With help of the Table 1, we deduce that $x_{n}\to \text{0}.\text{5}\text{0}\text{0}\text{0}\text{0}.$ This confirms the applicability of Theorem 4.7.

**Table 1:** The first five iterates of $\left\{x_{n}\right\}$ for different initial values.

Item	$x_{0}=0.10000$	$x_{0}=0.30000$	$x_{0}=0.70000$	$x_{0}=0.90000$
1	0.64007	0.57000	0.42996	0.35993
2	0.47828	0.48910	0.51086	0.52172
3	0.50109	0.50050	0.49946	0.49891
4	0.50002	0.50000	0.49999	0.49998
5	0.50000	0.50000	0.50000	0.50000

## 5. Applications

Ethics statement. This study did not involve human participants, animals, identifiable personal data, or human-derived materials. Therefore, ethical approval and informed consent were not required.

In this section, we present the results of experiments conducted to evaluate the performance of the proposed two-step G−I− nonexpansive iterative framework in image and signal restoration tasks. Image deblurring experiments were conducted using a set of widely used benchmark images. Signal restoration results are presented separately in Section 5.2. All methods were implemented in MATLAB R2016a under the same experimental conditions.

**Fig 1:**
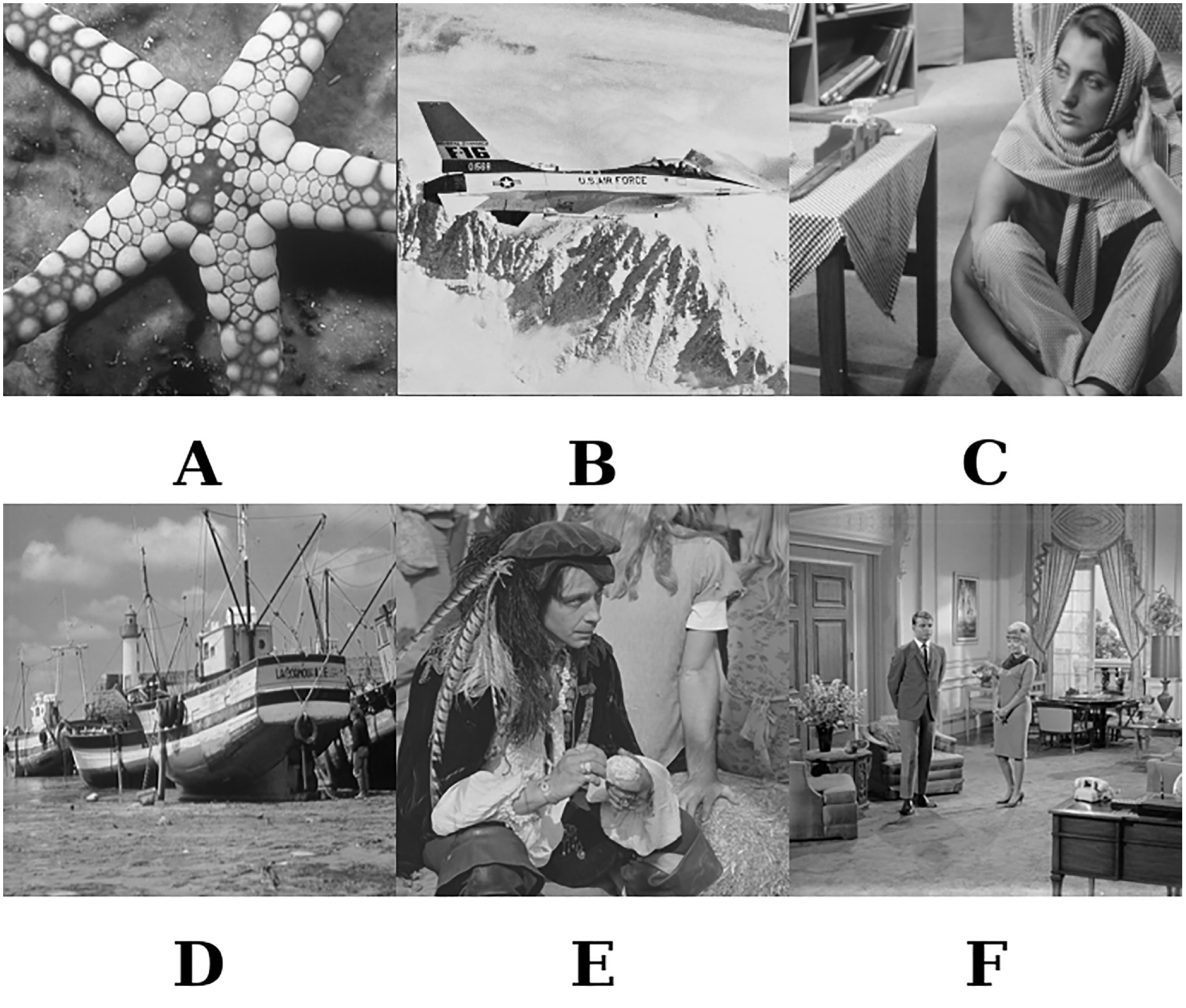
Original benchmark images used in the experiments: (A) Starfish, (B) Plane, (C) Woman, (D) Boats, (E) Pirate, and (F) Couple. The benchmark images are from the publicly available Set12 dataset available on Kaggle under the CC0 Public Domain license.

Fig 1 shows the original images used in the experiments. These images -- Starfish, Plane, Woman, Boats, Pirate and Couple -- cover a broad range of structures and textures, providing a comprehensive basis for performance evaluation. The images were selected from the widely used Set12 benchmark dataset, which is publicly available on Kaggle under the CC0 Public Domain license.

### 5.1. Experimental evaluation on benchmark images

Each image was degraded using a motion blur model with a point spread function (PSF), which was generated using linear motion of a length of 15 pixels and an angle of ${\text{0}}^{\circ }$. This was followed by the addition of Gaussian noise with a variance of 0.001. The blur operation was implemented using circular boundary conditions. These degraded images were then used as the initial inputs for all restoration methods. To ensure reproducibility, a fixed random seed was used to generate the noise. The parameter values were selected through empirical tuning based on experimental performance, and were then examined further via sensitivity analysis. Detailed results can be found in the Supporting Information. The selected parameter values correspond to the stable regions identified in the peak signal-to-noise ratio (PSNR) and the structural similarity index measure (SSIM) response surfaces.

The restoration performance was assessed using the PSNR and the SSIM. All images were normalised to the range [0,1] and the PSNR was derived from the mean squared error, assuming a maximum pixel value of 1. SSIM values were computed using MATLAB’s built-in implementation, if available; otherwise, a simplified global SSIM formulation was used as a fallback.

The baseline methods were implemented as follows: Wiener filtering with MATLAB’s deconvwnr function, LR with 20 iterations, TV-based deblurring using an ADMM framework, and FISTA with quadratic Laplacian regularization.

As shown in Table 2, the proposed method outperforms Wiener filtering, LR and FISTA consistently across all test images, achieving PSNR values ranging from approximately 21.10 to 24.51 dB. While TV-based deblurring achieves the highest PSNR values overall, the proposed method consistently ranks as the second-best performer.

**Table 2:** PSNR (dB) comparison of different methods on benchmark images.

Image	Proposed	Wiener	LR	TV	FISTA
Starfish	21.98	11.02	15.66	23.56	20.16
Plane	21.09	11.35	16.20	22.62	20.06
Woman	21.99	10.83	14.94	22.71	19.36
Boats	24.19	10.67	15.09	25.66	20.08
Pirate	23.51	11.05	15.77	25.27	20.49
Couple	24.50	10.65	15.04	25.78	20.11

As shown in Table 3, the proposed method achieves SSIM values ranging from 0.52 to 0.62, outperforming Wiener, LR and FISTA again for all images. As with the PSNR results, TV produces the highest SSIM values, while the proposed method maintains the second-best performance. To improve visualisation of the quantitative performance differences between the methods under comparison, the PSNR and SSIM values are presented in Supporting information (S1 Fig and S2 Fig).

**Table 3:** SSIM comparison of different methods on benchmark images.

Image	Proposed	Wiener	LR	TV	FISTA
Starfish	0.62	0.12	0.27	0.71	0.44
Plane	0.60	0.13	0.25	0.74	0.38
Woman	0.52	0.09	0.17	0.61	0.31
Boats	0.58	0.06	0.14	0.69	0.29
Pirate	0.56	0.07	0.20	0.66	0.36
Couple	0.58	0.06	0.16	0.68	0.32

**Fig 2:**
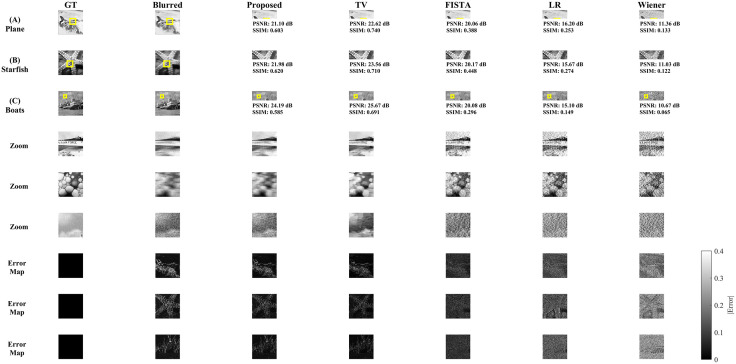
Visual comparison of restoration results on representative benchmark images: (A) Plane, (B) Starfish and (C) Boats. From left to right: ground truth; blurred and noisy observation; proposed method; total variation (TV); FISTA; LR; and Wiener filter. Zoomed-in regions and the corresponding error maps are shown below each example. PSNR (dB) and SSIM values are reported for each method. The original benchmark images (Plane, Starfish, and Boats) are from the publicly available Set12 dataset available on Kaggle (CC0 Public Domain license). All degraded images, restoration results, zoomed-in regions, error maps, and quantitative results were generated by the author using MATLAB.

Fig 2 shows typical restoration results. Wiener filtering produces poor results because the inversion of its frequency domain amplifies high-frequency noise components, particularly in regions with a low signal-to-noise ratio (SNR) where the denominator of the Wiener filter approaches zero. This results in severe noise amplification throughout the restored image. The LR algorithm improves local contrast and sharpness through the iterative back-projection of residual errors. However, ringing artefacts accumulate at sharp edges as iterations proceed due to the ill-posed nature of the deconvolution problem, which degrades the overall perceptual quality. TV-based restoration effectively suppresses noise and preserves edges, yielding the highest quantitative scores. However, it tends to oversmooth fine textures and produce regions of constant intensity, a limitation known as the staircasing effect. In contrast, the proposed method offers a stable, training-free alternative that balances noise suppression and structural preservation within an Ishikawa-type iterative framework. This approach does not require the parameter-intensive regularisation tuning associated with TV-based methods. Further qualitative results for the other test images can be found in Supporting Information (S3 Fig) to demonstrate that the visual behaviour is not confined to the examples given in Fig 2.

The Wiener filtering method amplifies noise in the frequency domain due to its inverse filtering mechanism [52]. The Lucy-Richardson method improves contrast and sharpness, but it can also introduce noise amplification and artefacts such as edge ringing, particularly when the number of iterations increases [53]. The TV-based method protects edges but does not restore them well, due to problems such as excessive smoothing and staircasing [54]. The proposed method combines data-fidelity correction (τ_data) with a smoothed regularisation (τ_reg, λ_reg) step within an Ishikawa-type iterative framework to reduce noise while preserving information. This is evaluated using PSNR and SSIM metrics on six test images.

The proposed restoration method follows a two-step Ishikawa-type iterative scheme. In the deblurring application, the abstract mapping C, is exemplified as the data-fidelity gradient step,


$$\begin{array}{c} Cx=x-\tau_{data}H^{T}(Hx-b), \end{array}$$

where H represents the blur operator, $H^{T}$ its adjoint operator, and b the distorted image. This step ensures consistency with the blurry, noisy image observed by reducing the discrepancy between the predicted and observed data. In the second step, the mapping I, which is instantiated as a smoothed Laplacian-based regularisation operator, is implemented:


$$\begin{array}{c} Iy=y-\tau_{reg}\;\lambda_{reg}\frac{Ly}{\sqrt{(Ly{)}^{\text{2}}+\varepsilon }}, \end{array}$$

where L represents the discrete Laplace operator, λ_reg the adjustment coefficient, and ε a small compensator constant introduced to improve numerical robustness. This step promotes spatial smoothness while preventing oversensitivity to local fluctuations. The final update combines these operators through convex combinations controlled by the parameters


$$\begin{array}{c} y=(\text{1}-\rho )x+\rho \text{Cx} \end{array}$$

$$\begin{array}{c} x_{k+\text{1}}=(\text{1}-\sigma \_I)x_{k}+Iy\sigma \_I. \end{array}$$

Thus, the practical application of image blur removal establishes a clear link between the theoretical formulation and the restoration algorithm by directly materialising the abstract fixed-point frame via mappings *C* and *I*.

The parameter ρ controls the contribution of the data-fidelity step within the Ishikawa iteration, whereas σ_I determines the influence of the regularisation update on the final estimate. The step sizes τ_data and τ_reg govern the gradient descent updates for the data-fidelity and regularization terms, respectively. The regularization parameter λ_reg balances noise suppression and structural preservation.

The parameter selection process involved two stages. In the first stage, the step sizes τ_data and τ_reg were set to0.8 and 0.15, respectively. These values were selected empirically to ensure stable convergence. The clipping operation x=min(max(x,0),1), which was applied at each iteration, further enforced stability by constraining the solution to the valid intensity range. In the second stage, the regularization parameters ρ and σ_I were determined through a structured grid search over ρ∈{0.2,0.4,0.6,0.8,1.0} and σ_I∈{0.1,0.2,0.3,0.4,0.5}, forming a 5 × 5 search space of 25 configurations evaluated on all six test images. The configuration ρ = 0.6 and $\sigma_{I}=\text{0}.\text{3}$ was observed to provide strong performance across all images and was selected as the final parameter setting. The regularization coefficient was fixed at λ_reg = 0.01 and the iteration count was set to maxIter=80.

**Fig 3:**
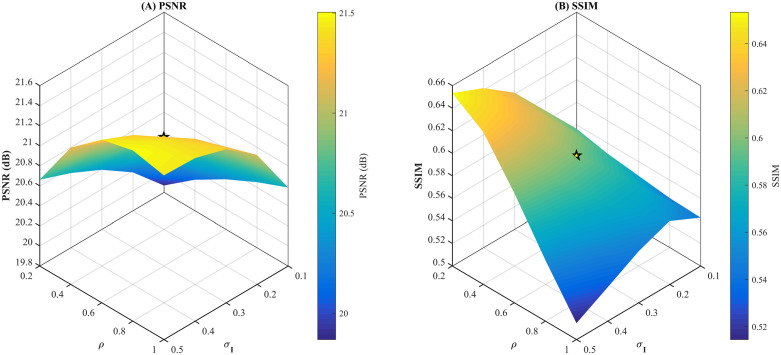
Sensitivity analysis of the proposed method on the Plane image. (A) Variation of PSNR and SSIM with respect to the parameter ρ. (B) Variation of PSNR and SSIM with respect to σ_I.

The results demonstrate a trade-off between reconstruction accuracy and structural preservation and confirm that the chosen parameter values deliver balanced performance. As shown in Fig 3, PSNR values increase with ρ, while SSIM decreases. This indicates a trade-off between reconstruction accuracy and structural preservation. Similarly, PSNR values increase with σ_I, while SSIM first increases and then decreases slightly after reaching its maximum at moderate values (approximately 0.3–0.4). These results suggest that moderate parameter values provide the best balance between noise suppression and detail preservation. The smooth variation of PSNR and SSIM across the tested ranges indicates that the method is stable and not overly sensitive to parameter selection. Based on these observations, the parameters ρ = 0.6 and $\sigma_{I}=\text{0}.\text{3}\;$were selected, as these provide a balanced trade-off between reconstruction accuracy and structural fidelity. The sensitivity analysis is illustrated using the Plane image, but similar trends were observed across other test images. Additional 2D sensitivity curves are provided in the Supporting Information (S4 Fig).

These findings demonstrate that the two-operator Ishikawa iteration is a robust and competitive solution for image restoration under Gaussian blur and noise. Future work will investigate additional blur types such as defocus and spatially varying blur, and explore adaptive step-size strategies. Broader comparisons will also be conducted with modern iterative and deep learning-based restoration techniques.

### 5.2. Simulation results for 1D signal enhancement

The two-step G−I− nonexpansive mapping proposed was evaluated numerically using a 1D signal enhancement problem. In the experimental setup, a synthetic test signal of length 1024 was blurred using a Gaussian kernel, after which it was degraded by additive Gaussian noise with a standard deviation of σ=0.01. The algorithm was implemented in MATLAB R2016 and executed using the recommended parameter settings. To enhance computational efficiency, an early stopping criterion based primarily on the lack of further PSNR improvement was employed.

The iterative process terminated at the 201*st* iteration due to the early stopping condition. The best performance was achieved at the 193*rd* iteration, with a PSNR value of 29.73*dB*. The final structural similarity index (SSIM) was approximately 0.8562.

**Fig 4:**
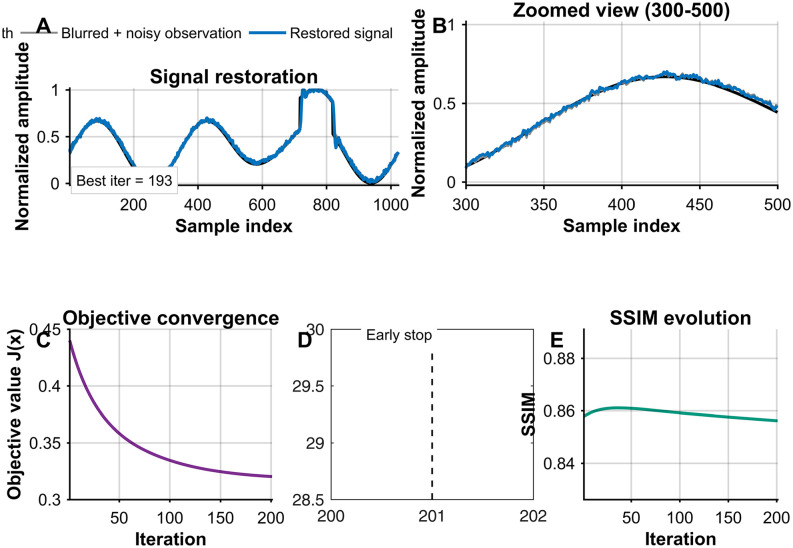
Convergence behaviour of the proposed two-step G-I-nonexpansive mapping under Gaussian blur and additive Gaussian noise (σ=0.01). (A) shows a comparison of the ground-truth signal, the blurred and noisy observation and the restored signal (best iteration = 193). (B) A zoomed view of the restoration process for samples 300–500. (C) Evolution of the objective function, showing a steady decrease. (D) PSNR (dB) progression with respect to the iteration number. The best value is attained at iteration 193 and the vertical dashed line indicates the iteration at which early stopping is initiated (201). (E) Evolution of the SSIM, which initially increases and then stabilises at a high level.

Fig 4 illustrates the convergence behaviour of the algorithm. It can be seen that the objective function steadily decreases over the iterations while the PSNR generally improves, reaching its maximum at the 193*rd* iteration. The SSIM initially improves, then exhibits slight variations and remains at a high level throughout the iterative process.

These results demonstrate that the proposed method effectively enhances signals degraded by blur and noise, providing stable and reliable convergence in terms of quantitative performance metrics.

## 6. Conclusion

In this work, inspired by the notion of g− edge preserving of C as introduced by the authors [26], we proposed the concept of G−I− nonexpansive mappings, which integrates the ideas of I−nonexpansive maps with graph-theoretic structures. Several convergence theorems of the iterative scheme (1) were established for G− nonexpansive mappings on abstract spaces under appropriate control conditions. These results extend and generalise the findings of Tripak [14]. To validate the theoretical findings, numerical experiments were conducted on benchmark image deblurring problems, involving images degraded by motion blur and additive Gaussian noise. The proposed method demonstrated robust and stable performance, consistently outperforming classical methods such as Wiener filtering, the LR and FISTA, while producing results comparable to those of TV-based approaches. Additionally, the method exhibited reliable convergence behaviour in 1D signal enhancement tasks, achieving high PSNR and SSIM values. These results confirm that the proposed iterative framework is an effective and versatile tool for signal and image restoration. Unlike deep learning methods, which are data- and resource-intensive, this approach requires no training and provides stable performance. Future research could involve extending the method to colour and high-resolution images. It could also examine how the method performs under different blur kernels and noise models. Furthermore, broader comparisons could be made with optimisation- and deep learning-based restoration techniques.

## Supporting information

S1 Fig PSNR comparison across benchmark images. This figure provides a visual representation of the quantitative results reported in Table 2, allowing clearer comparison of performance differences among the evaluated methods.
(PNG)

S2 Fig SSIM comparison across benchmark images. This figure provides a visual representation of the quantitative results reported in Table 3, enabling clearer comparison of structural similarity across methods.
(PNG)

S3 Fig Additional qualitative comparisons for the remaining test images. (PNG)

S4 Fig Sensitivity analysis results for different test images, illustrating the effect of parameter variations on PSNR and SSIM performance. (PNG)
