# Supplementary figures and images for: A two-step iterative framework for signal and image deblurring using G-I-Nonexpansive Mappings
Source: PLoS One. 2026 Jul 22;21(7):e0353844. doi: 10.1371/journal.pone.0353844 (PMC13390876; doi:10.1371/journal.pone.0353844)

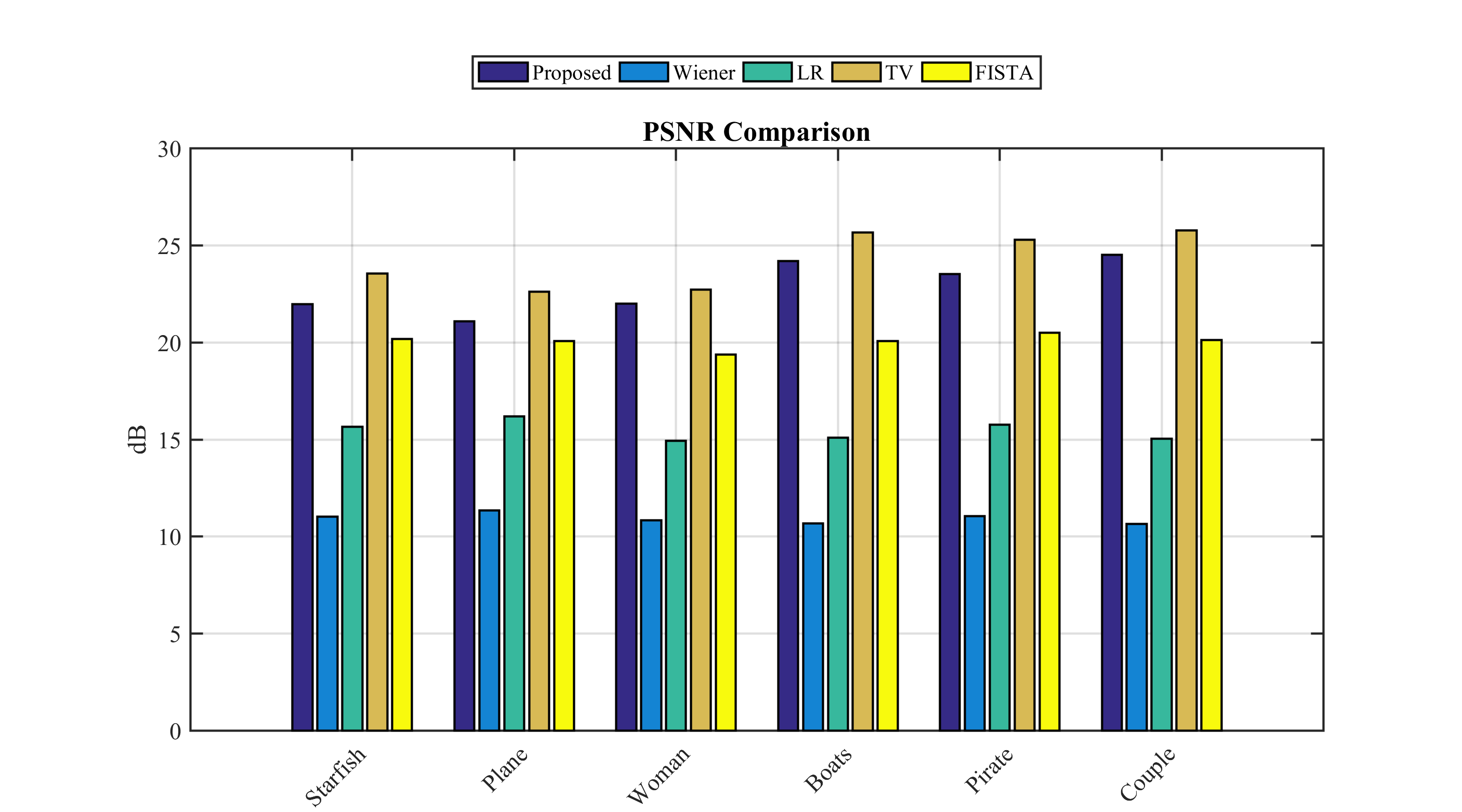

Supplement: S1 Fig — This figure provides a visual representation of the quantitative results reported in Table 2, allowing clearer comparison of performance differences among the evaluated methods. (PNG) [file pone.0353844.s001.png]

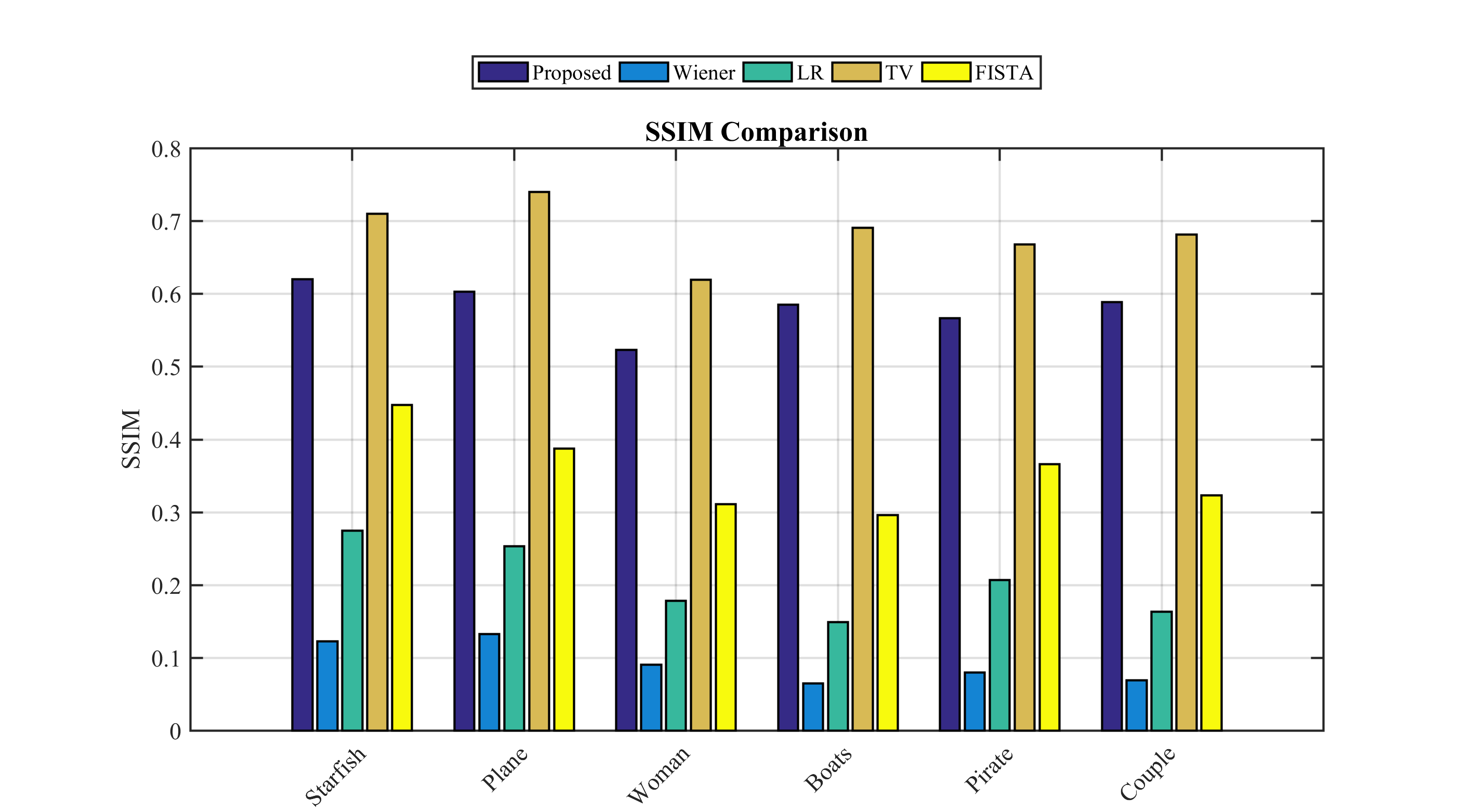

Supplement: S2 Fig — This figure provides a visual representation of the quantitative results reported in Table 3, enabling clearer comparison of structural similarity across methods. (PNG) [file pone.0353844.s002.png]

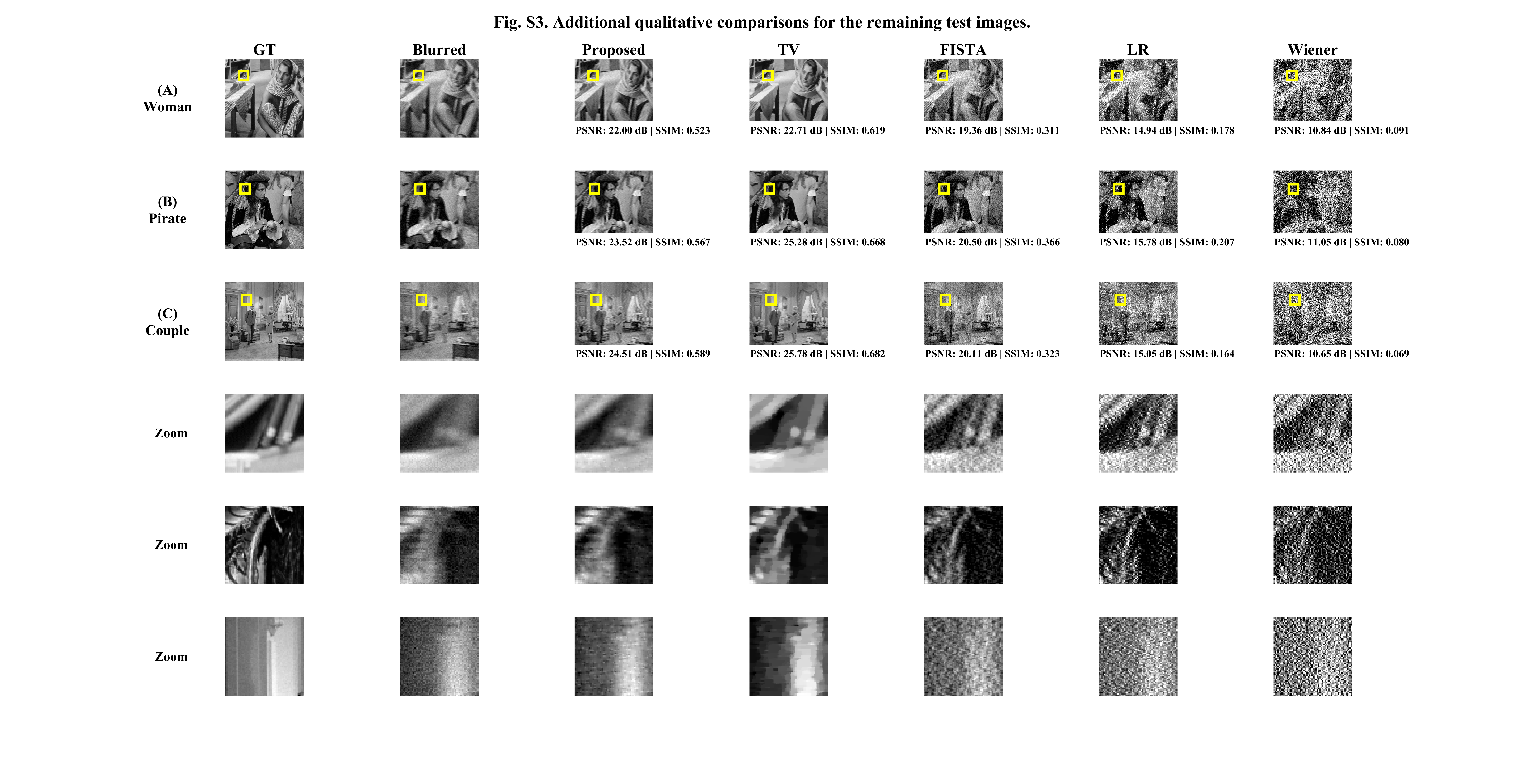

Supplement: S3 Fig — (PNG) [file pone.0353844.s003.png]

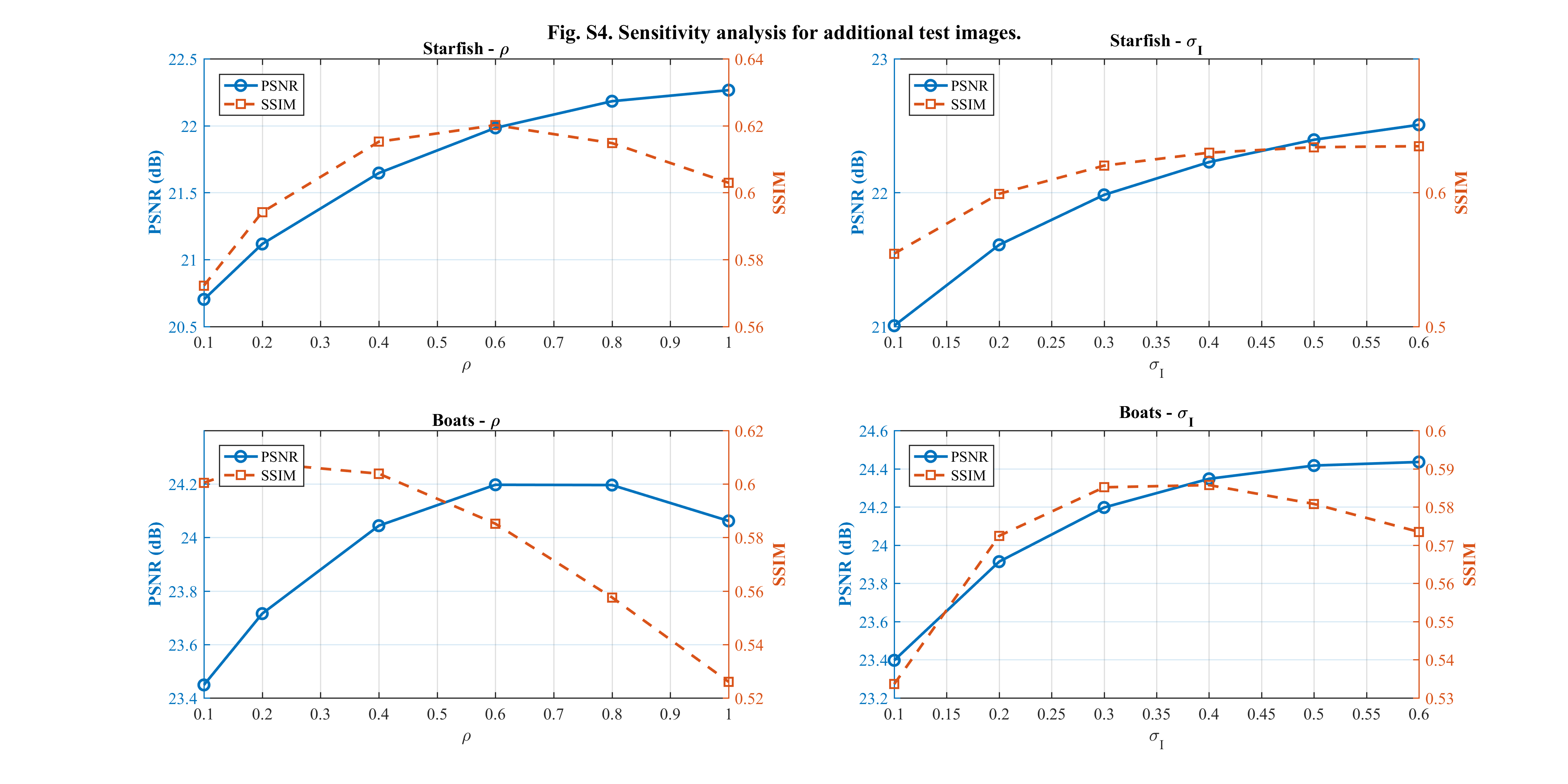

Supplement: S4 Fig — (PNG) [file pone.0353844.s004.png]
